# Modified primary tumour/vessel tumour/nodal tumour classification for patients with invasive ductal carcinoma of the breast

**DOI:** 10.1038/bjc.2011.279

**Published:** 2011-08-02

**Authors:** T Hasebe, M Iwasaki, S Akashi-Tanaka, T Hojo, T Shibata, Y Sasajima, T Kinoshita, H Tsuda

**Affiliations:** 1 Pathology Consultation Service, Clinical Trials and Practice Support Division, Center for Cancer Control and Information Services, National Cancer Center, 5-1-1, Tsukiji, Chuo-ku, Tokyo 104-0045, Japan; 2 Epidemiology and Prevention Division, Research Center for Cancer Prevention and Screening, National Cancer Center, 5-1-1, Tsukiji, Chuo-ku, Tokyo 104-0045, Japan; 3 Department of Breast Surgery, National Cancer Center Hospital, 5-1-1, Tsukiji, Chuo-ku, Tokyo 104-0045, Japan; 4 Cancer Genomics Project, National Cancer Center Research Institute, 5-1-1, Tsukiji, Chuo-ku, Tokyo 104-0045, Japan; 5 Clinical Laboratory Division, National Cancer Center Hospital, 5-1-1, Tsukiji, Chuo-ku, Tokyo 104-0045, Japan

**Keywords:** invasive ductal carcinoma, lymph vessel, blood vessel, lymph node, Nottingham Prognostic Index, prognosis

## Abstract

**Background:**

We previously reported that the primary tumour/vessel tumour/nodal tumour (PVN) classification is significantly superior to the UICC pTNM classification and the Nottingham Prognostic Index for accurately predicting the outcome of patients with invasive ductal carcinoma of the breast in a manner that is independent of the nodal status and the hormone receptor status.

**Methods:**

The purpose of the present study was to compare the outcome predictive power of a modified PVN classification to that of the newly devised pathological UICC pTNM classification and the reclassified Nottingham Prognostic Index in a different group of patients with invasive ductal carcinoma (*n*=1042) using multivariate analyses by the Cox proportional hazard regression model.

**Results:**

The modified PVN classification clearly exhibited a superior significant power, compared with the other classifications, for the accurate prediction of tumour recurrence and tumour-related death among patients with invasive ductal carcinoma in a manner that was independent of the nodal status, the hormone receptor status, and adjuvant therapy status.

**Conclusion:**

The modified PVN classification is a useful classification system for predicting the outcome of invasive ductal carcinoma of the breast.

We previously reported that the primary tumour/vessel tumour/nodal tumour (PVN) classification is significantly superior to the UICC pTNM classification (Sobin and Wittekind, 2002), the Nottingham Prognostic Index (Todd *et al*, 1987; Sundquist *et al*, 1999), and the histologic grade (Elston and Ellis, 1991) for accurately predicting the outcome of patients with invasive ductal carcinoma of the breast in a manner that is independent of the nodal status and the hormone receptor status (Hasebe *et al*, 2005). Since then, we newly devised a histological prognostic system, namely a grading system for lymph vessel tumour emboli, and have clearly demonstrated that this grading system can accurately predict the outcome of patients with invasive ductal carcinoma in a manner that is independent of nodal metastasis (Hasebe *et al*, 2008, 2010). In addition, although we have already reported that the diameter of the fibrotic focus is an important histological factor for predicting the outcome of patients with invasive ductal carcinoma without nodal metastasis (Hasebe *et al*, 1998, 2002a), this parameter was also found to be an important outcome predictor for patients with invasive ductal carcinoma with nodal metastasis in a study with a different patient group (Hasebe *et al*, 2009). Although we have separately devised a PVN classification based on histological factors, for example, the diameter of the fibrotic focus, the number of apoptotic figures of lymph vessel tumour emboli, and the number of invaded lymph vessels, for accurately predicting the outcome of patients with invasive ductal carcinoma with or without nodal metastasis (Hasebe *et al*, 2005), the above-mentioned studies strongly suggested that the factors used in this classification can accurately predict the outcome of patients with invasive ductal carcinoma in a manner that is independent of the nodal status. Thus, we attempted to refine the PVN classification using well-known histological factors as well as the factors that we proposed by analysing the outcome predictive powers of these factors in a different invasive ductal carcinoma patient group.

The purpose of this study was to compare the outcome predictive power of the modified PVN classification with that of the newly devised pathological UICC pTNM classification (Sobin *et al*, 2009), and the reclassified Nottingham Prognostic Index (Blamey *et al*, 2007) in a different group of patients with invasive ductal carcinoma. The latter two classifications are the major histological prognostic classifications currently in use clinically to predict the outcome of patients with breast carcinoma. The results clearly indicated that the modified PVN classification is a useful histological classification available for predicting the outcome of invasive ductal carcinoma of the breast in a manner that is independent of the nodal status, the hormone receptor status, and the adjuvant therapy status.

## Patients and methods

### Patients

The subjects of this study were 1042 consecutive patients with invasive ductal carcinoma of the breast who did not receive neoadjuvant therapy and were selected among 1759 patients with breast cancer who were surgically treated at the National Cancer Center Hospital between January 2000 and December 2005 (almost the same case series as that used in our previous study)(Hasebe *et al*, 2010). The invasive ductal carcinomas were diagnosed preoperatively using a needle biopsy, aspiration cytology, a mammography, or ultrasonography. All the patients investigated in this study were Japanese women, ranging in age from 23 to 72 years old (median, 55 years). All had a solitary lesion; 498 patients were pre-menopausal and 544 were post-menopausal. A partial mastectomy had been performed in 458 patients, and a modified radical mastectomy had been performed in 584. A level I and level II axillary lymph node dissection had been performed in all the patients, and a level III axillary lymph node dissection had been performed in some of the invasive ductal carcinoma patients.

Of the 1042 patients, 873 received adjuvant therapy, consisting of chemotherapy in 217 patients, endocrine therapy in 281 patients, and chemoendocrine therapy in 375 patients. The chemotherapy regimens used were anthracycline-based with or without taxane and non-anthracycline-based, and the endocrine therapy regimens consisted of tamoxifen with or without a gonadotropin-releasing hormone agonist, tamoxifen, with or without an aromatase inhibitor, an aromatase inhibitor alone, or a gonadotropin-releasing hormone agonist alone. No cases of inflammatory breast cancer were included in this series. All the tumours were classified according to the pathological UICC TNM classification (Sobin *et al*, 2009). The protocol for this study (20–112) was reviewed by the institutional review board of the National Cancer Center.

For the pathological examination, the surgically resected specimens were fixed in 10% formalin, and the size and gross appearance of the tumours were recorded. The tumour size was confirmed by comparison with the tumour size on the histological slides.

### Improvement of the PVN classification

We previously reported that a grading system for lymph vessel tumour emboli is a very useful histological classification in the accurate prediction of the outcome of patients with invasive ductal carcinoma independent of nodal status using multivariate analyses with well-known clinicopathological factors (Hasebe *et al*, 2008, 2010). Furthermore, the diameter of the fibrotic focus has also been reported to be a useful histological predictor of outcome for invasive ductal carcinoma patients with or without nodal metastasis using multivariate analyses with well-known clinicopathological factors (Hasebe *et al*, 2009). Thus, based on these findings, we attempted to improve our original PVN classification (Hasebe *et al*, 2005) by performing multivariate analyses using the Cox proportional hazard regression model to analyse the effects of well-known histological factors, our proposed histological factors, age (⩽39 *vs* >39 years), the Allred scores for oestrogen receptor and progesterone receptor and the category of HER2 expression in the tumour cells, and the type of adjuvant therapy (no adjuvant therapy, endocrine therapy, chemoendocrine therapy, and chemotherapy). Factors that were significantly associated with outcome in univariate analyses were entered together into a multivariate analysis performed using the Cox proportional hazard regression model. The case-wise, step-down, and two-sided method was applied until all the remaining factors were significant at a *P*-value of <0.05. All analyses were performed using Statistica/Windows software (StatSoft, Tulsa, OK, USA).

The following 12 histological factors of the primary invasive ductal carcinomas were evaluated: (1) invasive tumour size (⩽20 mm, >20 to ⩽50 mm, and >50 mm), (2) tubule formation (well degree, moderate degree, and poor degree) (Elston and Ellis, 1991), (3) number of mitotic figures in the primary invasive ductal carcinoma (⩽9, >9 to ⩽19, and >20) (Elston and Ellis, 1991), (4) nuclear features (small and regular, moderate variation, and marked variation) (Elston and Ellis, 1991), (5) tumour necrosis (absent and present)(Gilchrist *et al*, 1993), (6) blood vessel invasion (absent and present), (7) adipose tissue invasion (absent and present), (8) skin invasion (absent and present), (9) muscle invasion (absent and present), (10) fibrotic focus (absent, fibrotic focus diameter ⩽8 mm, and fibrotic focus diameter >8 mm)(Hasebe *et al*, 1998, 2002a), (11) grading system for lymph vessel tumour emboli (grades 0, 1, 2, and 3) (Hasebe *et al*, 2008, 2010), and (12) number of apoptotic figures in blood vessel tumour emboli (blood vessel invasion absent, ⩽2, and >2) (Hasebe *et al*, 2003a).

**Figure 1:**
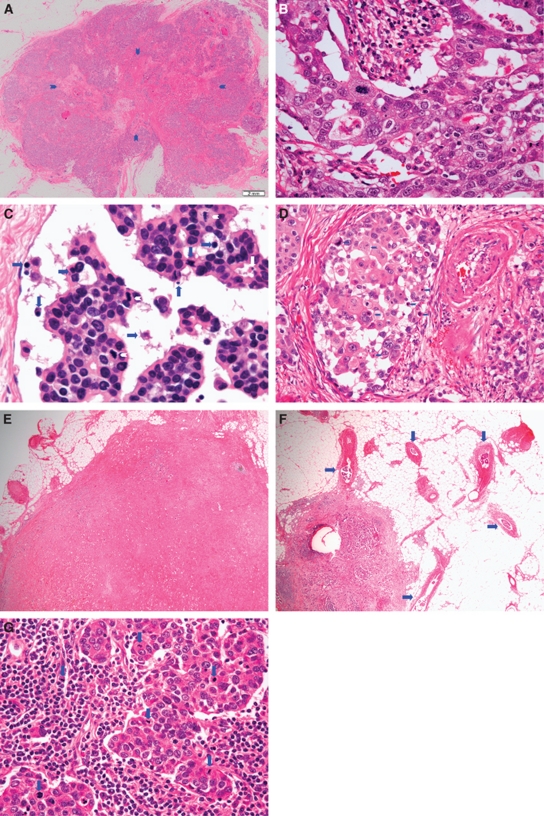
Histological factors of the modified PVN classification (**A**–**G**). (**A**) Invasive ductal carcinomas with a fibrotic focus. A fibrotic focus measuring 13.4 × 6.8 mm is visible within the tumour (panoramic view, arrows). The fibrotic focus shows a scar-like feature and is surrounded by invasive ductal carcinoma cells. (**B**) Invasive ductal carcinoma cells showing marked variations in nuclear features; mitotic figures are also visible in the tumour cells. (**C**) Several apoptotic bodies and apoptotic tumour cells are visible (arrows), and three mitotic tumour cells (arrowheads) are visible in the tumour embolus in the lymph vessel. (**D**) One blood vessel tumour embolus is seen adjacent to one artery. Seven apoptotic bodies or apoptotic tumour cells are seen in the blood vessel tumour embolus, and red blood cells are also visible. (**E**) Lymph node metastases exhibited a severe grade of tumour stroma. (**F**) Five extranodal blood vessel tumour emboli are seen in metastatic carcinoma to the lymph node (arrows). (**G**) Six mitotic tumour cells are visible in the tumour of the lymph node (arrows).

The nodal metastases were evaluated using single sections of each node or half of each node stained with hematoxylin and eosin. The following 10 histological parameters of metastatic mammary carcinoma to the lymph nodes were evaluated: (1) number of nodal metastases (no nodal metastasis, 1–3, 4–9, 10, or more), (2) maximum dimension (no nodal metastasis, ⩽20 mm, and >20 mm), (3) tubule formation (no nodal metastasis, well degree, moderate degree, and poor degree), (4) nuclear features (no nodal metastasis, small and regular, moderate variation, and marked variation), (5) number of mitotic figures (no nodal metastasis, ⩽5, and >5)(Hasebe *et al*, 2003b, 2004, 2011), (6) fibrotic focus (no nodal metastasis, absent, and present), (7) tumour necrosis (no nodal metastasis, absent, and present), (8) grade of stromal fibrosis (no nodal metastasis, none, mild, moderate, and severe) (Hasebe *et al*, 2003b, 2004, 2011), (9) number of lymph nodes with extranodal invasion (no nodal metastasis, ⩽5, and >5) (Hasebe *et al*, 2003b, 2004, 2011), and (10) number of extranodal blood vessel tumour emboli (no nodal metastasis, <2, and >2) (Hasebe *et al*, 2003b, 2004, 2011). We randomly searched for mitotic figures in metastatic mammary carcinoma to the lymph nodes using mid-power magnification fields ( × 10 or × 20) of the tumour area and selected one high-power magnification field ( × 40) of the tumour area with the highest number of mitotic figures in metastatic mammary carcinoma to the lymph nodes to determine the largest number of metastatic mammary carcinoma to the lymph nodes exhibiting mitotic figures (Figure 1). The tubule formation, nuclear features, and presence of tumour necrosis in metastatic mammary carcinoma to the lymph nodes were evaluated in the same manner as for the primary invasive ductal carcinomas.

### Prognostic histological classifications for comparative study

The following existing histological classifications were compared with our modified classification with regard to the prediction of disease-free survival and overall survival: (1) the UICC pTNM classification (Sobin *et al*, 2009) and (2) the Nottingham Prognostic Index (Blamey *et al*, 2007).

The Nottingham Prognostic Index (Blamey *et al*, 2007) is based on the tumour size, histologic grade (Elston and Ellis, 1991), and nodal classification of the UICC pTNM classification (Sobin *et al*, 2009). Thus, multivariate analyses for tumour recurrence and tumour-related death were performed using the following models to avoid the mutual influences of each classification: (1) model 1, the modified PVN classification and the UICC pTNM classification; and (2) model 2, the modified PVN classification and the Nottingham Prognostic Index.

The predictive powers for the disease-free and overall survivals of each classification, age (⩽39 *vs* >39 years), the Allred scores for oestrogen receptor and progesterone receptor and the category of HER2 expression in the tumour cells, and the type of adjuvant therapy (no adjuvant therapy, endocrine therapy, chemoendocrine therapy, and chemotherapy) were evaluated using univariate analyses with the Cox proportional hazard regression model according to nodal status, hormone receptor status, and adjuvant therapy status. The classifications and factors that were significantly associated with outcome in the univariate analyses were then entered together into the multivariate analyses that were performed using the Cox proportional hazard regression model. The case-wise, step-down and two-sided method was applied until all the remaining factors were significant at a *P*-value of <0.05. The survival curves were drawn using the Kaplan–Meier method. All the analyses were performed with Statistica/Windows software (StatSoft).

### Assessment of oestrogen receptor, progesterone receptor, and HER2 expression

Slides of the tumour cells immunostained for oestrogen receptor or progesterone receptor were scored using the Allred scoring system, as described previously (Harvey *et al*, 1999; Mohsin *et al*, 2004), and the Allred scores for oestrogen receptor and progesterone receptor in the tumour cells were classified into the following three categories (Hasebe *et al*, 2009): (1) Allred score for oestrogen receptor in tumour cells (0 or 2, 3–6, and 7 or 8); and (2) Allred score for progesterone receptor in tumour cells (0 or 2, 3–6, and 7 or 8). The HER2 status of the tumour cells was semiquantitatively scored on a scale of 0–3 according to the level of HER2 protein expression (Wolff *et al*, 2007) and was classified into three categories: 0 or 1, 2, and 3.

### Patient outcome

Survival was evaluated using a median follow-up period of 98 months (range: 63–134 months) until March 2011. Of the 1042 invasive ductal carcinoma patients, 858 patients were alive and well, 184 had developed tumour recurrences (distant-organ metastasis and local recurrence), and 89 had died of their disease. The tumour recurrence-free survival and overall survival periods were calculated using the time of surgery as the starting point. Tumour relapse was considered to have occurred whenever evidence of distant-organ metastasis or local recurrence was found.

## Results

### Histological factors significantly associated with the outcome of patients with invasive ductal carcinoma

Among all the patients with invasive ductal carcinoma, a fibrotic focus diameter >8 mm, lymph vessel tumour embolus grades 2 and 3, lymph node metastases with a dimension of >20 mm, >2 apoptotic figures in blood vessel tumour emboli, and >5 mitotic figures in metastatic carcinoma to the lymph nodes had significantly higher hazard ratios for tumour recurrence and tumour-related death in multivariate analyses (Table 1). Marked variation in the nuclear features of the primary invasive ductal carcinoma had a significantly higher hazard ratio for tumour recurrence, and >2 extranodal blood vessel tumour emboli had a significantly higher hazard ratio for tumour-related death in multivariate analyses (Table 1). An Allred score of 7 or 8 for progesterone receptor in the tumour cells had a significantly lower hazard ratio for tumour-related death in multivariate analysis (Table 1).

Among patients with invasive ductal carcinoma without nodal metastasis, lymph vessel tumour grade 3 and >19 mitotic figures in primary invasive ductal carcinomas had a significantly higher hazard ratio for tumour recurrence and tumour-related death in multivariate analyses (Table 2). Lymph vessel tumour grade 2, a fibrotic focus >8 mm, and HER2 category 3 had significantly higher hazard ratios for tumour recurrence, and >2 apoptotic figures in blood vessel tumour emboli had a significantly higher hazard ratio for tumour-related death in multivariate analyses.

Among patients with invasive ductal carcinoma with nodal metastases, a fibrotic focus diameter >8 mm, lymph vessel tumour embolus grades 2 and 3, lymph node metastases with a dimension of >20 mm, >2 apoptotic figures in blood vessel tumour emboli, >2 extranodal blood vessel tumour emboli, and >5 mitotic figures in metastatic carcinoma to the lymph nodes had significantly higher hazard ratios for tumour recurrence and tumour-related death in multivariate analyses (Table 3). A severe grade of stromal fibrosis in metastatic carcinoma to the lymph nodes had a significantly higher ratio for tumour recurrence, and an Allred score of 7 or 8 for progesterone receptors had a significantly lower hazard ratio for tumour-related death in a multivariate analysis (Table 3).

### Modified PVN classification

**Figure 2:**
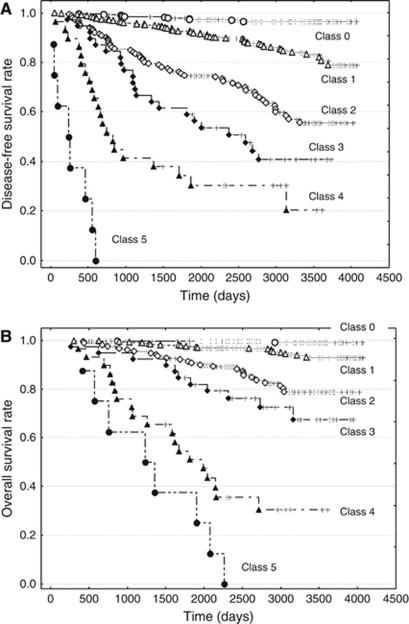
Disease-free survival curve and overall survival curve according to the modified PVN classification for all the patients in the present study (**A** and **B**). The disease-free survival curve (**A**) and the overall survival curve (**B**) for each class significantly decrease according to the increasing order of the classifications (*P*<0.001).

We refined our previously proposed PVN classification (Hasebe *et al*, 2005) based on the above results of the present study, and the nine parameters that were selected for modified PVN classification are listed in Table 4 (Figure 1). Four factors (mitotic figures in primary invasive ductal carcinoma, lymph vessel tumour grade, grade of stromal fibrosis in metastatic carcinoma to the lymph nodes, and maximum dimension of metastatic carcinoma to the lymph nodes) were newly added to the classification based on the results of this study. In the modified PVN classification, a score of 1 point was given for each unfavourable parameter. A grading system of 0–3 was used to score the lymph vessel tumour emboli (Hasebe *et al*, 2008, 2010) (Figure 1D). The total score was then calculated (0–11). The resulting scores were divided into the following six classes according to their significant associations with tumour recurrence or tumour-related death in univariate analyses using the logrank test: (1) class 0 (score 0), (2) class 1 (scores 1 and 2), (3) class 2 (scores 3 and 4), (4) class 3 (score 5), (5) class 4 (scores 6 and 7), and (6) class 5 (score of 8 or more) (Table 5, Figure 2).

Furthermore, we also attempted to modify the PVN classification using the weight given to each factor based on its median hazard ratio obtained using the multivariate analyses in this study. The weights that were given for each factor were as follows: (1) 2.0 for a fibrotic focus diameter >8 mm, (2) 1.5 for the marked variation of nuclear features of primary invasive ductal carcinoma, (3) 7.2 for >19 mitotic figures in primary invasive ductal carcinoma, (4) 2.9 for lymph vessel tumour embolus grade 2 and 4.2 for lymph vessel tumour embolus grade 3, (5) 3.2 for >2 apoptotic figures in blood vessel tumour emboli, (6) 1.9 for a severe grade of stromal fibrosis in metastatic carcinoma to the lymph nodes, (7) 1.9 for lymph node metastases with a dimension of >20, (8) 1.9 for >2 extranodal blood vessel tumour emboli, and (9) 3.1 for >5 mitotic figures in metastatic carcinoma to the lymph nodes. The total factor weights for individual patients were calculated (minimum, 0; maximum, 27.3; median, 1.5) and we classified all the patients into the following five classes based on the total factor weight for each patient: (1) 484 patients with a total factor weight of 0, class 0; (2) 192 with a total factor weight of >0 to ⩽5, class 1; (3) 321 with a total factor weight of >5 to ⩽10, class 2; (4) 31 with a total factor weight of >10 to ⩽18, class 3; and (5) 14 with a total factor weight of >18, class 4.

Next, we performed multivariate analyses for tumour recurrence and tumour-related death between the score-modified PVN and the weight-modified PVN classification as a whole. Although both classifications significantly increased the hazard ratios for tumour recurrence and tumour-related death, the score-modified PVN classification (tumour recurrence: trend hazard ratio, 2.1, *P*<0.001; tumour-related death: trend hazard ratio, 2.2, *P*<0.001) had higher trend hazard ratios and lower trend *P*-values for tumour recurrence and tumour-related death than the weight-modified PVN classification (tumour recurrence: trend hazard ratio, 1.3, *P*=0.019; tumour-related death: trend hazard ratio, 1.5, *P*=0.033). Therefore, we adopted the former classification for a comparison with the other two classifications in this study.

### Tumour recurrence and death rates according to each classification

According to the modified PVN classification, the rates of tumour recurrence or death from invasive ductal carcinoma increased as the order of the classes increased; the rates of classes 4 and 5, in particular, were higher than those of the high-risk groups of the other classifications (Table 5). Significantly shorter crude disease-free survival and overall survival periods were observed according to the increasing order of classes, with the exception of both survival periods for classes 3 and 4 and the overall survival periods for classes 4 and 5 (Table 5, Figure 2).

Among the other classifications, the UICC pTNM stage classification showed significantly shorter crude disease-free survival and overall survival periods according to the increasing order of stages (Table 5). Among the three classifications, the Nottingham Prognostic Index clearly exhibited the lowest tumour recurrence rate in patients with a good prognosis (excellent prognostic group). The Nottingham Prognostic Index showed a significantly shorter crude disease-free survival period according to the increasing order of groups with the exception of moderate prognostic group II, but significant differences in the overall survival periods were seen between the moderate prognostic group II and the poor prognostic group, and between the poor prognostic group and the very poor prognostic group out of the six groups (Table 5).

### Comparison of the classifications

In model 1 multivariate analyses of all the patients, the modified PVN classification significantly increased the trend hazard ratios for tumour recurrence (*P*<0.001) and tumour-related death (*P*<0.001). Although the UICC pTNM classification showed a significant association with tumour recurrence (*P*=0.018), it failed to show a significant association with tumour-related death (*P*=0.165). HER2 category 3 had a significant association with tumour recurrence (*P*=0.033). In model 2 multivariate analyses, the modified PVN classification significantly increased the trend hazard ratios for tumour recurrence (*P*<0.001) and tumour-related death (*P*<0.001). The Nottingham Prognostic Index also showed significant associations with tumour recurrence (*P*=0.003) and tumour-related death (*P*=0.006). HER2 category 3 failed to significantly increase the hazard ratio for tumour recurrence in model 2 multivariate analyses.

In patients with invasive ductal carcinoma without nodal metastasis, the UICC pTNM classification failed to show a significant association with tumour recurrence or tumour-related death in univariate analyses (data not shown). In model 1 multivariate analyses, the modified PVN classification was significantly associated with tumour recurrence (*P*<0.001) and tumour-related death (*P*<0.001). In model 2 multivariate analyses, the modified PVN classification was significantly associated with tumour recurrence and tumour-related death, but the Nottingham Prognostic Index was not significantly associated with tumour recurrence or tumour-related death (Table 6).

In patients with invasive ductal carcinoma with nodal metastasis, the modified PVN classification showed significant associations with tumour recurrence and tumour-related death but the UICC pTNM classification did not show a significant association with tumour recurrence or tumour-related death in model 1 multivariate analyses (Table 6). In model 2 multivariate analyses, the modified PVN classification also showed significant associations with tumour recurrence and tumour-related death. The Nottingham Prognostic Index did not show a significant association with tumour recurrence, but a significant association with tumour-related death was observed (Table 6).

In patients with invasive ductal carcinoma who were completely negative for hormone receptors, only the modified PVN classification showed significantly increasing trend hazard ratios for tumour recurrence and tumour-related death in the multivariate analyses (Table 6).

In model 1 and 2 multivariate analyses of patients with invasive ductal carcinoma who were positive for one or two hormone receptors, the modified PVN classification exhibited significantly increasing trend hazard ratios for tumour recurrence and tumour-related death (Table 6). The Nottingham Prognostic Index also showed significantly increasing trend hazard ratios for tumour recurrence and tumour-related death (Table 6). Although the UICC pTNM classification significantly increased the trend hazard ratio for tumour recurrence, it failed to significantly increase the trend hazard ratio for tumour-related death (Table 6). In model 1 and 2 multivariate analyses, the adjuvant therapy status significantly increased the trend hazard ratios for tumour-related death (model 1, *P*=0.007; model 2, *P*=0.022) but failed to significantly increase the trend hazard ratios for tumour recurrence (model 1, *P*=0.996; model 2, *P*=0.597).

In model 1 and 2 multivariate analyses of patients with invasive ductal carcinoma not treated with adjuvant therapy, the modified PVN classification significantly increased the hazard ratios for tumour recurrence (Table 7). The UICC pTNM classification and the Nottingham Prognostic Index failed to show significant associations with tumour recurrence (Table 7). HER2 category 3 significantly increased the trend hazard ratio for tumour recurrence in a model 1 multivariate analysis (*P*=0.048) but failed to significantly increase the trend hazard ratio for tumour recurrence in a model 2 multivariate analysis (*P*=0.093). As only five patients died as a result of their disease in this series, a multivariate analysis for tumour-related death could not be performed.

In model 1 and 2 multivariate analyses of patients with invasive ductal carcinoma treated with endocrine therapy, the modified PVN classification significantly increased the trend hazard ratios for tumour recurrence and tumour-related death (Table 7). The UICC pTNM classification and the Nottingham Prognostic Index failed to show significant associations with tumour recurrence and tumour-related death (Table 7). In model 1 and 2 multivariate analyses, HER2 category 3 significantly increased the trend hazard ratios for tumour-related death (model 1 and model 2, *P*<0.001) but failed to significantly increase the trend hazard ratios for tumour recurrence (model 1, *P*=0.082; model 2, *P*=0.086).

In model 1 and 2 multivariate analyses of patients with invasive ductal carcinoma treated with chemoendocrine therapy, the modified PVN classification significantly increased the hazard ratios for tumour recurrence and tumour-related death (Table 7). The UICC pTNM classification did not show significantly increasing trend hazard ratios for tumour recurrence and tumour-related death (Table 7). The Nottingham Prognostic Index significantly increased the trend hazard ratios for tumour recurrence and tumour-related death (Table 7).

In model 1 and 2 multivariate analyses of patients with invasive ductal carcinoma treated with chemotherapy, although the modified PVN classification significantly increased the trend hazard ratios for tumour recurrence and tumour-related death, the UICC pTNM classification and the Nottingham Prognostic Index failed to show significant associations with tumour recurrence or tumour-related death (Table 7).

## Discussion

We previously reported that the PVN classification can accurately predict the outcome of patients with invasive ductal carcinoma in a manner that is independent of the nodal status or hormone receptor status (Hasebe *et al*, 2005), and the present study also clearly demonstrated that the modified PVN classification accurately predicted the outcome of patients with invasive ductal carcinoma in a manner that was independent of the nodal status, hormone receptor status, or adjuvant therapy status in a different group of patients with invasive ductal carcinoma. The clinical value of prognostic factors is particularly useful for the selection of different treatment regimens, especially adjuvant therapy in patients with invasive ductal carcinoma. One could argue that identifying patients with invasive ductal carcinoma who have a good prognosis and who do not require adjuvant therapy is of particular importance. The modified PVN classification was capable of classifying 815 (78%) out of 1042 patients as class 0 or 1, and patients belonging to these classes may be considered as good and moderately good prognostic groups, respectively. In contrast, patients belonging to class 2 or higher classes of the modified PVN classification may be considered as belonging to poor or very poor prognostic groups, respectively. In addition, the modified PVN classification had a superior outcome predictive power for the other two classifications in a manner that was independent of the adjuvant therapy status. Thus, the results of this study suggest that patients belonging to class 0 or 1 of the modified PVN classification can be spared adjuvant therapy, while patients belonging to class 2 or higher classes of the classification should be treated with adjuvant therapy in a manner that is independent of the nodal status or the hormone receptor status.

The factors included in the modified PVN classification were selected based on the precise analyses of this study using well-known clinicopathological factors, such as histologic grade, invasive tumour size, and nodal status. Among the nine factors in the modified PVN classification, seven of them were the histological factors that we proposed for primary invasive ductal carcinoma, carcinomas in vessels, and metastatic carcinoma to the lymph nodes (Hasebe *et al*, 1998, 2002a, 2003a, 2003b, 2004, 2008, 2010, 2011). This study clearly confirmed that these histological factors are important outcome predictors for different patient series of invasive ductal carcinoma of the breast. Among them, the outcome predictive power of the fibrotic focus among patients with invasive ductal carcinoma has also been confirmed by other investigators (Colpaert *et al*, 2001; Baak *et al*, 2005). Thus, these parameters are likely to be the most suitable parameters for accurately assessing the true biological malignant potential of invasive ductal carcinomas. In addition, we also confirmed the prognostic significance of the following factors that were previously reported by other investigators (Elston and Ellis, 1991) to be useful histological factors for predicting the outcome of patients with invasive ductal carcinomas: (1) the nuclear features of primary invasive ductal carcinoma and (2) the number of mitotic figures in primary invasive ductal carcinoma. Thus, the modified PVN classification appears to be better at accurately predicting the outcome of patients with invasive ductal carcinoma, compared with the other two classifications.

This study also strongly suggests that the tumour characteristics of invasive ductal carcinomas matter more than the quantity of tumour with regard to the accurate prediction of the outcome of patients with invasive ductal carcinoma. Both the UICC pTNM stage classification and the Nottingham Prognostic Index evaluate the malignant potential of invasive ductal carcinomas based on the invasive tumour size and the number of nodal metastases. These factors reflect the quantity of invasive ductal carcinoma cells. In contrast, almost all the factors in the modified PVN classification, exception of the maximum diameter of lymph node metastases, represent the tumour characteristics of invasive ductal carcinomas. In addition, we previously showed that mitotic figures and apoptotic figures in tumour cells of lymph vessel tumour emboli have significantly stronger outcome predictive powers than the number of lymph vessels that have been invaded (Hasebe *et al*, 2002b), and we devised a grading system for lymph vessel tumour emboli based on the presence of mitotic figures and apoptotic figures in the tumour cells of lymph vessel tumour emboli (Hasebe *et al*, 2008, 2010). As the modified PVN classification can evaluate the tumour characteristics of the invasive ductal carcinoma more precisely than the other two classifications, it appears to have a superior ability for accurately predicting patient outcome. Therefore, we concluded that the modified PVN classification is a useful prognostic histological classification available for predicting the outcome of patients with invasive ductal carcinoma of the breast.

We used the modified PVN classification for patients with invasive ductal carcinoma because our previous studies clearly demonstrated that the factors included in this classification were significant outcome predictors only for patients with invasive ductal carcinoma (Hasebe *et al*, 1998, 2002a, 2003a, 2003b, 2004, 2008, 2010, 2011). The UICC pTNM classification and the Nottingham Prognostic Index can be applied to all invasive breast carcinomas and may be superior to the modified PVN classification for predicting the outcome of overall patients with invasive carcinoma. Thus, we should confirm whether the modified PVN classification is also able to accurately predict the outcome of patients with non-ductal carcinomas of the breast in the future.

In conclusion, the current study clearly confirmed that the modified PVN classification is a useful histological classification for predicting the outcome of patients with invasive ductal carcinoma of the breast. Thus, pathologists should attempt to assess the true malignant potential of invasive ductal carcinomas using the criteria of the modified PVN classification.

## Figures and Tables

**Table 1:** Multivariate analyses for tumour recurrence and tumour-related death in all the invasive ductal carcinoma patients (*n*=1042)

	**Tumour recurrence**				**Tumour-related death**		
	**Cases**	**Cases (%)**	**HR (95% CI)**	***P*-value**	**Cases (%)**	**HR (95% CI)**	***P*-value**
*Fibrotic focus, diameter (mm)*							
Absent	667	95 (14)	Referent		42 (6)	Referent	
⩽8	221	37 (17)	1.0 (0.7–1.5)	0.914	15 (7)	1.0 (0.5–1.9)	0.999
>8	154	52 (34)	1.9 (1.4–2.6)	<0.001	32 (21)	2.2 (1.3–3.6)	0.003
							
*Grading system for lymph vessel tumour emboli*							
Grade 0	666	74 (11)	Referent		30 (5)	Referent	
Grade 1	250	43 (17)	1.6 (0.9–2.6)	0.074	18 (7)	1.4 (0.8–2.5)	0.308
Grade 2	97	46 (47)	3.5 (2.4–5.1)	<0.001	24 (25)	2.9 (1.6–5.2)	<0.001
Grade 3	29	21 (72)	4.7 (2.8–8.0)	<0.001	17 (59)	3.1 (1.5–6.3)	0.002
							
*Maximum dimension of metastatic carcinoma to the lymph nodes (mm)*							
no	591	54 (9)	Referent		17 (3)	Referent	
⩽20	396	102 (26)	Referent		53 (13)	Referent	
>20	55	28 (51)	1.8 (1.1–2.7)	0.011	19 (35)	1.9 (1.0–3.7)	0.040
							
*Number of apoptotic figures in blood vessel tumour emboli*							
Absent	890	138 (16)	Referent		61 (7)	Referent	
⩽2	78	15 (19)	2.6 (0.3–19.5)	0.359	6 (8)	2.6 (0.3–19.5)	0.359
>2	74	21 (42)	2.9 (2.0–4.4)	<0.001	22 (30)	3.2 (1.8–5.6)	<0.001
							
*Number of mitotic figures in metastatic carcinoma to lymph nodes*							
n0	591	54 (9)	Referent		17 (3)	Referent	
⩽5	283	46 (16)	Referent		17 (6)	Referent	
>5	165	84 (51)	2.6 (1.8–3.7)	<0.001	55 (33)	3.6 (2.1–6.6)	<0.001
							
*Nuclear feature of primary invasive tumours*							
Small	27	1 (4)	Referent		1 (4)	Referent	
Mod	770	101 (13)	1.5 (0.2–10.9)	0.697	38 (5)	0.3 (0.04–2.5)	0.265
Marked	245	82 (33)	1.5 (1.1–2.1)	0.012	50 (20)	1.3 (0.5–3.0)	0.576
							
*Allred scores for progesterone receptors in primary invasive tumour cells*							
0 or 2	183	48 (26)	Referent		24 (13)	Referent	
3–6	303	58 (19)	0.9 (0.5–1.5)	0.553	36 (12)	1.3 (0.6–2.6)	0.518
7 or 8	556	78 (14)	0.9 (0.5–1.5)	0.717	29 (5)	0.5 (0.3–0.8)	0.007
							
*Number of extranodal blood vessel tumour emboli*							
no	591	54 (9)	Referent		17 (3)	Referent	
⩽2	423	17 (4)	Referent		11 (3)	Referent	
>2	28	19 (68)	1.5 (0.8–2.8)	0.256	18 (65)	1.9 (1.0–3.6)	0.036

Abbreviations: HR=hazard ratio; CI=confidence interval; no=no nodal metastasis; small=small and regular in size; mod=moderate variation; marked=marked variation.

**Table 2:** Multivariate analyses for tumour recurrence and tumour-related death in invasive ductal carcinoma patients without nodal metastases

**Patients without nodal metastasis (*n*=591)**							
	**Tumour recurrence**				**Tumour-related death**		
	**Cases**	**Cases (%)**	**HR (95% CI)**	***P*-value**	**Cases (%)**	**HR (95% CI)**	***P*-value**
*Grading system for lymph vessel tumour emboli*							
Grade 0	465	38 (8)	Referent		11 (2)	Referent	
Grade 1	111	9 (8)	0.9 (0.4–1.8)	0.690	4 (4)	1.6 (0.5–5.2)	0.474
Grade 2	14	6 (43)	5.9 (2.4–14.2)	<0.001	1 (7)	2.4 (0.3–20.8)	0.437
Grade 3	1	1 (100)	42.8 (5.3–344.6)	<0.001	1 (100)	96.0 (5.9–1545.7)	0.001
							
*Number of mitotic figures in primary invasive tumours (/10 high-power fields)*							
⩽9	285	12 (4)	Referent		1 (0.4)	Referent	
>9–⩽19	153	15 (10)	1.4 (0.6–3.2)	0.467	5 (3)	6.6 (0.7–60.1)	0.093
>19	153	27 (18)	2.0 (1–3.5)	0.025	11 (7)	12.4 (1.2–125.5)	0.035
							
*Fibrotic focus, diameter (mm)*							
Absent	415	33 (8)	Referent		11 (3)	Referent	
⩽8	114	8 (7)	0.8 (0.3–1.8)	0.571	2 (2)	0.5 (0.1–3.2)	0.476
>8	62	13 (21)	2.3 (1.2–4.6)	0.011	4 (7)	1.1 (0.3–4.4)	0.908
							
*HER 2 category in primary invasive tumour cells*							
0 or 1	424	28 (7)	Referent		8 (2)	Referent	
2	104	12 (12)	1.3 (0.6–3.1)	0.483	4 (4)	1.6 (0.4–7.6)	0.526
3	63	14 (22)	2.0 (1.1–3.9)	0.032	5 (8)	2.7 (0.7–10.2)	0.138
							
*Number of apoptotic figures in blood vessel tumour emboli*							
Absent	528	46 (9)	Referent		14 (3)	Referent	
⩽2	33	3 (9)	1.1 (0.3–3.6)	0.916	0	Referent	
>2	30	5 (17)	2.0 (0.7–5.3)	0.175	3 (10)	4.1 (1.1–16.0)	0.041

Abbreviations: HR=hazard ratio; CI=confidence interval.

**Table 3:** Multivariate analyses for tumour recurrence and tumour-related death in invasive ductal carcinoma patients with nodal metastases

**Patients with nodal metastases (*n*=451)**							
	**Tumour recurrence**				**Tumour-related death**		
	**Cases**	**Cases (%)**	**HR (95% CI)**	***P*-value**	**Cases (%)**	**HR (95% CI)**	***P*-value**
*Fibrotic focus, diameter (mm)*							
Absent	252	62 (25)	Referent		31 (12)	Referent	
⩽8	107	29 (27)	1.5 (0.8–2.8)	0.175	13 (12)	1.0 (0.5–2.2)	0.945
>8	92	39 (42)	1.6 (1.1–2.4)	0.020	28 (30)	2.0 (1.2–3.3)	0.005
							
*Grading system for lymph vessel tumour emboli*							
Grade 0	201	36 (18)	Referent		19 (10)	Referent	
Grade 1	139	34 (25)	1.6 (0.9–2.7)	0.083	14 (10)	1.1 (0.5–2.5)	0.740
Grade 2	83	40 (48)	2.6 (1.7–3.9)	<0.001	23 (28)	2.8 (1.6–5.0)	<0.001
Grade 3	28	20 (71)	3.4 (1.9–6.0)	<0.001	16 (57)	3.6 (1.9–7.1)	<0.001
							
*Maximum dimension of metastatic carcinoma to the lymph nodes (mm)*							
⩽20	396	102 (26)	Referent		53 (13)	Referent	
>20	55	28 (51)	1.6 (1.0–2.4)	0.044	19 (35)	2.0 (1.1–3.8)	0.029
							
*Number of apoptotic figures in blood vessel tumour emboli*							
Absent	362	92 (25)	Referent		47 (13)	Referent	
⩽2	45	12 (27)	1.5 (0.2–12.9)	0.693	6 (13)	9.7 (0.9–111.1)	0.065
>2	24	26 (59)	3.1 (1.9–4.9)	<0.001	19 (43)	3.2 (1.9–5.6)	<0.001
							
*Number of extranodal blood vessel tumour emboli*							
⩽2	423	111 (26)	Referent		54 (13)	Referent	
>2	28	19 (68)	1.8 (1.1–3.2)	0.034	18 (65)	2.1 (1.1–3.8)	0.019
							
*Number of mitotic figures in metastatic carcinoma to lymph nodes*							
⩽5	286	46 (16)	Referent		17 (6)	Referent	
>5	165	84 (51)	2.7 (1.8–3.9)	<0.001	55 (33)	3.4 (1.9–6.2)	<0.001
							
*Grade of stromal fibrosis in metastatic mammary carcinoma to the lymph nodes*							
None	101	19 (19)	Referent		9 (9)	Referent	
Mild	177	38 (22)	0.8 (0.4–1.4)	0.354	20 (11)	0.6 (0.2–1.6)	0.330
Mod	137	54 (39)	1.2 (0.6–2.1)	0.666	32 (23)	1.0 (0.4–2.6)	0.999
Severe	36	19 (53)	1.9 (1.2–3.2)	0.009	11 (31)	0.7 (0.2–2.4)	0.554
							
*Allred scores for progesterone receptors in primary invasive tumour cells*							
0 or 2	79	33 (42)	Referent		18 (23)	Referent	
3–6	134	40 (30)	0.8 (0.5–1.4)	0.442	30 (22)	1.1 (0.5–2.2)	0.862
7 or 8	238	57 (24)	0.9 (0.5–1.6)	0.704	24 (10)	0.5 (0.3–0.9)	0.010

Abbreviations: HR=hazard ratio; CI=confidence interval; mod=moderate variation.

**Table 4:** Parameters of the modified primary tumour/vessel tumour/nodal tumour classification for patients with invasive ductal carcinoma of the breast

**Parameters**	**Scores**
1. Fibrotic focus, diameter, in primary invasive tumours	
Absent/⩽ 8 mm *vs* >8 mm	0 *vs* 1
2. Nuclear feature of primary invasive ductal carcinomas	
Small/moderate *vs* marked	0 *vs* 1
3. Number of mitotic figures in primary invasive ductal carcinomas (/10 high-power fields)	
⩽19 *vs* >19	0 *vs* 1
4. Grading system for lymph vessel tumour emboli	
Grades 0, 1, 2, and 3	0–3
5. Number of apoptotic figures in blood vessel tumour emboli	
Absent/⩽2 *vs* >2	0 *vs* 1
6. Grade of stromal fibrosis in metastatic mammary carcinoma to the lymph nodes	
n0/none/mild/moderate *vs* severe	0 *vs* 1
7. Maximum dimension of metastatic carcinoma to the lymph nodes (mm)	
n0/⩽20 *vs* >20	0 *vs* 1
8. Number of extranodal blood vessel tumour emboli	
n0/⩽2 *vs* >2	0 *vs* 1
9. Number of mitotic figures in metastatic carcinoma to the lymph nodes	
n0/⩽5 *vs* >5	0 *vs* 1
	
	Total 0–11

Abbreviation: no=no metastatic tumour.

**Table 5:** Tumour recurrence and death rates according to the modified primary tumour/vessel tumour/nodal tumour classification, the UICC pTNM stage classification, and the Nottingham Prognostic Index among all the patients with invasive ductal carcinoma (*n*=1042)

**Primary tumour/vessel tumour/nodal tumour classification**					
**Classes (scores)**	**Cases**	**TRR (%)**	***P*-value**	**MR (%)**	***P*-value**
Class 0 (0)	349	11 (3)		2 (0.6)	
Class 1 (1/2)	466	66 (14)	<0.001	23 (5)	0.018
Class 2 (3/4)	151	56 (37)	0.005	26 (17)	0.002
Class 3 (5)	39	22 (56)	0.001	11 (28)	0.030
Class 4 (6/7)	29	21 (72)	0.390	19 (66)	0.505
Class 5 (8–11)	8	8 (100)	0.047	8 (100)	0.111
					
*UICC pTNM stage classification*					
Stage I (IA and IB)	352	26 (7)		9 (3)	
Stage II (IIA and IIB)	494	87 (18)	<0.001	34 (7)	0.004
Stage IIIA and IIIB	148	42 (28)	0.003	25 (17)	<0.001
Stage IIIC	48	29 (60)	<0.001	21 (44)	<0.001
					
*Nottingham Prognostic Index*					
Excellent prognostic group	130	1 (0.8)		0	
Good prognostic group	240	15 (6)	0.015	3 (1)	0.235
Moderate prognostic group I	252	38 (15)	0.002	10 (4)	0.069
Moderate prognostic group II	240	45 (19)	0.175	23 (10)	0.009
Poor prognostic group	118	48 (41)	<0.001	23 (19)	0.009
Very poor prognostic group	62	37 (60)	0.007	30 (48)	<0.001
					
Total	1042	169		67	

Abbreviations: TRR=tumour recurrence rate; MR=mortality rate.

**Table 6:** Multivariate analyses for disease-free and overall survival for the modified primary tumour/vessel tumour/nodal tumour classification, the UICC pTNM stage classification, and the Nottingham Prognostic Index in patients with invasive ductal carcinoma according to nodal status or hormone receptor status

	**Disease-free survival**		**Overall survival**	
**Classifications**	**Trend HR (95% CI)**	**Trend *P*-value**	**Trend HR (95% CI)**	**Trend *P*-value**
*Patients with invasive ductal carcinoma without nodal metastasis (*n=*592)*				
Model 2				
PVN (0–5)	2.1 (1.3–3.5)	0.003	3.4 (1.5–7.7)	0.004
NPI (EPG, GPG, MPGI, MPGII, PPG, VPG)	1.4 (0.9–2.1)	0.065	1.3 (0.7–2.5)	0.449
				
*Patients with invasive ductal carcinoma with nodal metastasis (*n=*450)*				
Model 1				
PVN (0–5)	2.2 (1.9–2.5)	<0.001	2.4 (1.9–2.9)	<0.001
pTNM (I, II, IIIAB, IIIC)	1.2 (0.9–1.5)	0.180	1.2 (0.9–1.7)	0.232
Model 2				
PVN (0–5)	2.2 (1.8–2.6)	<0.001	2.1 (1.7–2.7)	<0.001
NPI (EPG, GPG, MPGI, MPGII, PPG, VPG)	1.1 (0.9–1.4)	0.259	1.5 (1.1–2.0)	0.024
				
*Patients with invasive ductal carcinoma who were completely negative for hormone receptors (*n=*125)*				
Model 1				
PVN (0–5)	2.3 (1.6–3.3)	<0.001	2.6 (1.7–4.3)	<0.001
pTNM (I, II, IIIAB, IIIC)	1.3 (0.8–2.1)	0.344	1.3 (0.6–2.6)	0.548
Model 2				
PVN (0–5)	2.5 (1.7–3.6)	<0.001	2.4 (1.5–4.1)	<0.001
NPI (EPG, GPG, MPGI, MPGII, PPG, VPG)	1.1 (0.7–1.6)	0.779	1.3 (0.7–2.5)	0.426
				
*Patients with invasive ductal carcinoma who were positive for one or two hormone receptors (*n=*917)*				
Model 1				
PVN (0–5)	2.3 (1.9–2.6)	<0.001	2.4 (2.0–3.0)	<0.001
pTNM (I, II, IIIAB, IIIC)	1.3 (1.0–1.6)	0.024	1.2 (0.9–1.6)	0.206
Model 2				
PVN (0–5)	2.0 (1.7–2.4)	<0.001	2.1 (1.6–2.7)	<0.001
NPI (EPG, GPG, MPGI, MPGII, PPG, VPG)	1.3 (1.1–1.6)	0.002	1.4 (1.1–1.9)	0.013

Abbreviations: HR=hazard ratio; CI=confidence interval; PVN=modified primary tumour/vessel tumour/nodal tumour; NPI=Nottingham Prognostic Index; EPG=excellent prognostic group; GPG=good prognostic group; MPGI=moderate prognostic group I; MPGII=moderate prognostic group II; PPG=poor prognostic group; VPG=very poor prognostic group; pTNM=UICC pTNM; IIIAB=UICC pTNM stages IIIA and IIIB.

**Table 7:** Multivariate analyses for disease-free and overall survival for the modified primary tumour/vessel tumour/nodal tumour classification, the UICC pTNM stage classification, and the Nottingham Prognostic Index in patients with invasive ductal carcinoma according to adjuvant therapy status

	**Disease-free survival**		**Overall survival**	
**Classifications**	**Trend HR (95% CI)**	**Trend (*P*-value)**	**Trend HR (95% CI)**	**Trend (*P*-value)**
*Patients with invasive ductal carcinoma not treated with adjuvant therapy (*n=*169)*				
Model 1				
PVN (0–5)	2.4 (1.4–4.1)	0.001	NA	
pTNM (I, II, IIIAB, IIIC)	1.2 (0.6–2.5)	0.653	NA	
Model 2				
PVN (0–5)	2.1 (1.2–3.7)	0.012	NA	
NPI (EPG, GPG, MPGI, MPGII, PPG, VPG)	1.5 (0.8–2.4)	0.120	NA	
				
*Patients with invasive ductal carcinoma treated with endocrine therapy (*n=*281)*				
Model 1				
PVN (0–5)	3.4 (2.5–4.8)	<0.001	5.6 (2.8–11.1)	<0.001
pTNM (I, II, IIIAB, IIIC)	1.3 (0.8–2.1)	0.291	0.5 (0.2–1.5)	0.205
Model 2				
PVN (0–5)	2.9 (1.9–4.5)	<0.001	4.7 (2.2–10.4)	<0.001
NPI (EPG, GPG, MPGI, MPGII, PPG, VPG)	1.3 (0.9–1.8)	0.128	0.8 (0.4–1.8)	0.662
				
*Patients with invasive ductal carcinoma treated with chemoendocrine therapy (*n=*375)*				
Model 1				
PVN (0–5)	2.0 (1.6–2.5)	<0.001	2.1 (1.5–3.0)	<0.001
pTNM (I, II, IIIAB, IIIC)	1.4 (0.9–1.9)	0.057	1.4 (0.9–2.3)	0.115
Model 2				
PVN (0–5)	1.7 (1.3–2.3)	<0.001	1.7 (1.1–2.7)	0.011
NPI (EPG, GPG, MPGI, MPGII, PPG, VPG)	1.4 (1.1–1.8)	0.012	1.6 (1.1–2.5)	0.020
				
*Patients with invasive ductal carcinoma treated with chemotherapy (*n=*217)*				
Model 1				
PVN (0–5)	2.1 (1.6–2.8)	<0.001	2.2 (1.7–2.8)	<0.001
pTNM (I, II, IIIAB, IIIC)	1.3 (0.9–2.0)	0.188	1.3 (0.9–1.8)	0.152
Model 2				
PVN (0–5)	2.3 (1.7–3.0)	<0.001	2.0 (1.5–2.7)	<0.001
NPI (EPG, GPG, MPGI, MPGII, PPG, VPG)	1.1 (0.8–1.5)	0.619	1.4 (0.9–2.1)	0.133

Abbreviations: HR=hazard ratio; CI=confidence interval; PVN=modified primary tumour/vessel tumour/nodal tumour; NPI=Nottingham Prognostic Index; EPG=excellent prognostic group; GPG=good prognostic group; MPGI=moderate prognostic group I; MPGII=moderate prognostic group II; PPG=poor prognostic group; VPG=very poor prognostic group; pTNM=UICC pTNM; IIIAB=UICC pTNM stages IIIA and IIIB; NA=not available.
